# The Economic Costs of Congenital Hearing Loss in a South African Cohort

**DOI:** 10.1177/11786329251401414

**Published:** 2026-03-19

**Authors:** Winfrida Tombe-Mdewa, Claudine Storbeck, Alys Young, Aisha Moolla, Susan Goldstein, Evelyn Thsehla

**Affiliations:** 1 Faculty of Health Sciences, SAMRC/WITS Centre for Decision Science and Health Economics (PRICELESS SA), Wits School of Public Health, University of Witwatersrand, Johannesburg, South Africa; 2 Centre for Deaf Studies, University of the Witwatersrand, Johannesburg, South Africa; 3 School of Nursing, Midwifery and Social Work, University of Manchester, UK

**Keywords:** congenital hearing loss, cost of illness, economic costs, societal costs, South Africa

## Abstract

**Background:**

The prevalence of congenital hearing loss in South Africa is estimated to be 6 per 1000 live births, which is higher than most high-income countries but consistent with rates observed in other parts of Africa. While policymakers typically focus on direct healthcare-related costs, many of the broader societal and economic impacts of congenital hearing loss remain hidden, including educational, vocational, and social consequences. Understanding the full economic burden is essential for informed policy and healthcare planning.

**Objectives:**

To assess the full economic burden of congenital hearing loss, including indirect and intangible costs in South Africa.

**Design:**

A cost-of-illness model from a societal perspective in 2022.

**Methods:**

We constructed a cohort-survival model to estimate the societal lifetime costs of congenital hearing loss. Cost components included direct medical expenses (diagnostics, devices, surgeries), non-medical costs (special education), indirect costs (lost productivity, reduced earnings) and the monetary value of disability adjusted life years lost due to hearing loss. Model inputs were derived from published literature and consultation with experts.

**Results:**

Total economic cost for the cohort was ZAR 16.4 billion (USD 1 billion per lifetime or ZAR 256 million (USD 15.9 million) per year. Productivity losses made up the largest proportion of societal costs with losses of approximately ZAR 2.4 billion (52% of total costs). Total discounted cost per person with hearing loss was ZAR 1.1 million (USD 70 thousand) per lifetime.

**Conclusion:**

Congenital hearing loss has far-reaching implications beyond immediate healthcare expenses, including significant societal and economic costs. Early childhood interventions such as newborn screening and early identification, assistive devices and speech and language therapy interventions may reduce the high costs associated with congenital hearing loss and may provide substantial returns on investment.

## Introduction

Hearing loss is the most prevalent sensory disability in childhood, disproportionately affecting approximately 4 to 6 per 1000 live births in developing countries annually compared with 1 to 2 per 1000 in developed countries.^1,2^ In South Africa, hearing loss amongst all ages is the third highest reported disability with reported prevalence estimates varying from 12.5%^3^ to 19.9%.^4^ It is estimated that approximately 6000 babies in South Africa are born with bilateral, permanent hearing loss annually.^5^ Congenital or early-onset sensorineural hearing loss typically occurs during the perinatal period—from the 20th week of gestation to the 28th day after birth and can be genetic, syndromic, chromosomal or acquired due to a range of aetiological risk factors and early childhood events.^6^ Early diagnosis, and interventions are essential to developmental outcomes in later childhood for children with congenital hearing loss.^7^

Early childhood hearing loss, if not identified early and concomitant early interventions put in place, will negatively affect early childhood development, cognitive and psycho-social development, educational attainment, and economic independence later in life.^8-10^ In 2017, an estimated additional USD 3.9 billion worldwide was required to provide education support among children aged 5 to 14 years with moderate to severe hearing loss.^11^ This is equivalent to 0.18% of GDP per capita. Approximately 73% of these costs are incurred by low- and middle-income countries (LMICs).^11^ Hearing loss is also associated with unemployment and underemployment of deaf individuals over their lifetime when compared to hearing individuals with the same qualifications.^12^

The rising global prevalence of hearing loss is costly both in terms of the health and well-being of those affected in addition to the financial losses arising from their exclusion from communication, education and employment.^11,13^ Global estimates of the economic costs of hearing loss (from all causes and at all ages) are more than USD $981 billion and 53% of these global costs are incurred in low and middle-income countries (LMICs).^11^ In 2015, unemployment and early retirement among adults with hearing loss resulted in productivity losses of USD 105 billion; 71% of which was incurred in LMICs.^11^ Intangible costs associated with social exclusion, language difficulties, and stigma were estimated to be USD 573 billion per annum.^11^

In South Africa, approximately 3.6 million South Africans of all ages require health care support for hearing loss of whom fewer than 10% are receiving any.^14^ Studies to date have addressed the prevalence, causes and the need for or feasibility of potential interventions to address hearing loss in adults and early intervention in infants and children in South Africa.^3,4,15–22^ There has not been any attempt to estimate the economic burden of hearing loss to South Africa and there is no information on the economic losses resulting from childhood hearing loss in South Africa.

We conducted a cost of illness study to ascertain the economic costs of congenital hearing loss in South Africa. Data on costs associated with congenital hearing loss could help inform the development of policies and inform resource allocation decisions for distribution of funds to support early identification and early intervention services, healthcare, education and employment programs.

## Methods

Using an Excel model, we adopted a bottom-up costing analysis which described the lifetime societal costs incurred by a cohort of individuals with congenital hearing loss.^23^ The study was conducted from a societal perspective. We estimated the direct medical, special education, social assistance costs, productivity losses and the monetary value of disability adjusted life years (DALYs) losses associated with congenital hearing loss from birth till death. A targeted literature review was conducted to identify cost components for the analysis. obtained through a literature search conducted on Google Scholar, Embase, Scopus and PubMed. Search terms included “hearing loss” or “hearing impairment” and “cost of illness” or “economic burden” or “healthcare costs”. Due to the paucity of literature on economic costs of hearing loss, we did not impose a time or geographic restriction. We further consulted subject matter experts to identify the cost components and to verify accuracy and contextual relevance of information extracted from the literature review. All costs were originally calculated in South African Rand (ZAR) and presented with United States dollar (USD) equivalents.

### Study Population

The study population was children born in 2022 with congenital hearing loss in South Africa.

### Prevalence

We estimated the cases of congenital hearing impairment in the base year by applying hearing loss prevalence rates to demographic information on the number of live births in South Africa in 2022 obtained from Statistics SA.^24,25^ Life expectancy for the cohort was 64 years.^26^

We then applied age-specific mortality rates obtained from the WHO life tables to determine survival of the cohort throughout their lifetime.^27^ We assumed that survival probability for individuals with hearing loss was the same as that of individuals without hearing loss.

### Direct Health Care Costs

Direct health care costs were estimated using a bottom-up ingredients-based approach. We used the Health Professions Council of South Africa newborn hearing screening protocol recommended in hospitals and clinics^28^ for screening costs. Literature review and key informant interviews were used to inform costs associated with the diagnosis and management of hearing loss including early intervention to support language development.

Assumptions were made where information was not available Supplemental File 1, Table 1. For the purposes of this analysis, the direct costs of hearing loss were those incurred within the public healthcare system by the South African government. Costs for screening, healthcare services utilization, the provision of amplification devices such as hearing aids and cochlear implants, aural rehabilitation and drugs were included. The costs of treating otitis media were also included. The SA government does not provide family centered early intervention as a routine provision for pre-school deaf. Early intervention costs therefore were not included other than those associated with hearing. Resource quantities and unit costs were obtained from published literature. Costs were adjusted for inflation using the Consumer Price Index (CPI).^29^ Future costs (cost incurred beyond the base year) were discounted at a rate of 0.05 as recommended by the South African pharmacoeconomic guidelines.^30^ Direct costs were estimated by multiplying unit costs by resource quantities.

**Table 1.** Model Parameters.

Parameters	Base case values	Source
Number of live births in SA in 2022	998 362	South African Government^34^
Prevalence of congenital hearing loss in SA	0.04	Chadha et al^10^
Levels of hearing loss classification		Storbeck et al^46^
Mild	4.51%	
Moderate	17.38%	
Severe	31.12%	
Profound	47%	
Probability of being screened at birth	0.1	Neumann et al^47^
Referrals for diagnostic testing after newborn screening	0.47	Bezuidenhout et al^48^
Proportion of infants referred for further screening after birth who are presented for further screening	0.35	Bezuidenhout et al^48^
Proportion of infants who are presented for further screening who require auditory brainstem response(ABR) testing	0.10	Bezuidenhout et al^48^
Number of specialist visits per year	4	Expert opinion
*Assistive device parameters*		
Hearing aid	R 10 197.41	South African Tender Price in Public Health Sector
Bilateral ear molds	R 694.85	South African National Treasury^49^
Batteries	R 45.58	Market Price
Cochlear implants	1 756 227.98	Kerr et al^50^
*Education cost parameters*		
Years of education	12	Assumption
Average public sign based residential education cost per annum	R 138 160.33	Emmett et al^31^
% of deaf children in mainstream schooling	0.85	Expert opinion
% of deaf children out of schools	0.15	Expert opinion
*Social assistance costs*		
Care dependency grant	1980	Trafford^51^
Adult disability grant	1980	Trafford^51^
% of population with self-care difficulties	0.05	Eckert et al^38^
% of moderate to profound hearing population that receives social support 0-14 years	7.2%	National Department of Social Development^52^
% of population with moderate to profound hearing loss that receives social support 15-64 years	31.5%	National Department of Social Development^52^
% of population with moderate to profound hearing population that receives social support above 59 years of age	38.5%	National Department of Social Development^52^
*Lost productivity estimates*		
GNI per capita		World Bank Group^53^
GNI per capita growth per annum	0.73%	Estimation
Life expectancy	64	Institute for Health Metrics and Evaluation^24^
Unemployment rate	33.50%	Drummond et al^42^
Discount rate	0.05	South African Department of Health^30^
Employed/population ratio	38.63	Drummond et al^42^
Labor force participation rate	58.08	Drummond et al^42^
*Monetary value of DALYs estimates*		
GDP per capita 2022	R 76 790.00	World Bank Group^54^
Current health expenditure 2022	R 9180.12	World Bank Group^55^
Rand/USD exchange rate in 2022	16.11	South African Reserve Bank^56^
CPI 2010	0.043	Statistics South Africa^57^
CPI 2012	0.057	Statistics South Africa^58^
CPI 2015	0.052	Statistics South Africa^59^
CPI 2020	0.033	Statistics South Africa^60^
CPI 2022	0.069	Statistics South Africa^61^

### Indirect Costs

The indirect costs of hearing loss reflect the economic impacts of hearing loss outside the healthcare system. The indirect costs of hearing loss considered in this analysis were financial costs associated with lower productivity from lower workforce participation in adulthood, additional costs incurred in the education sector, social assistance costs and the monetary value of DALYs lost due to hearing loss.

#### Education Costs

We estimated the cost of special schools for a duration of 12 years by applying cost per annum to the number of children attending and then discounting over 12 years. We assumed that 85% of deaf children in South Africa attend special schools. Average cost per annum estimates for special education were obtained from a previous study.^31^ The costs were converted to the South African rand. Cost estimates included staff teaching costs, facility costs and residential accommodation.

#### Social Assistance Costs

We estimated the total discounted lifetime cost of the disability and care dependency grants for the cohort. The South African disability grant (DG) is a non-contributory, means-tested cash transfer, available on a permanent or temporary basis to people deemed unfit to work due to a mental or physical disability. Recipients of the grant are South African citizens or residents aged between 18 and 59 years of age who pass a means income and asset test. In principle, the disability grant benefit amount is determined through a formula but in practice, a large majority of beneficiaries tend to receive the maximum benefit amount.^32^ The Care Dependency Grant (CDG) is the primary form of social protection available to caregivers of children with disabilities.^33^ It is given to a parent, primary caregiver or a foster parent of a child between birth to 18 years old who has a severe disability and is in need of full-time and special care.^34^ We applied the 2022 grant allocations to the eligible population and discounted the costs across their lifetime. The CDG is mainly accessed by families of children who have severe intellectual, physical or emotional impairments, or a combination of impairments or illnesses. For the purposes of this study, we assumed that all families in the cohort with children with self-care difficulties and are deaf would receive the CDG and that the child would go on to receive the DG after they turned 18.^35^ Data for this were obtained from the national census.^36,37^

#### Productivity Losses

Using Gross National Income per capita (GNI) we estimated productivity losses due to unemployment and reduced productivity for moderate to profound hearing loss using methods used in previous studies.^38,39^ We estimated that GNI per capita would grow by the average GNI per capita growth of South Africa between 2021 and 2023. We assumed that individuals were economically active from 15 until 64 years and thereafter would no longer contribute to productivity losses. Evidence shows that people who have disabilities in South Africa have higher chances of being chronically unemployed (unemployed for more than 12 months) or economically inactive but unemployment data estimates for people with hearing loss are unavailable in South Africa.^40^ We therefore assumed the same rate of unemployment as the general population and accounted for possible variation in the sensitivity analysis. Age specific unemployment rates and Labor Force Participation Rates (LFPRs) were applied in the model to account for age specific productivity levels in South Africa. Age group data on unemployment rates and LFPR were obtained from the Quarterly Labor Force Survey (QLFS) which is a household-based sample survey conducted by Statistics South Africa.^41^ A South African study found that there was a 57% wage differential between the non-disabled and disabled working population in South Africa.^40^ We estimated the working population by multiplying the LPFR and employment rate for each age group by the respective surviving population. Discounted productivity losses due to unemployment were estimated by multiplying the number of unemployed people in each age group by the GNI per capita and discounted. Productivity losses due to reduced productivity were estimated by applying the 0.57% economic factor to the number of employed people for each group and the GNI per capita and discounted.

#### Monetary Value of Disability Adjusted Life Years Lost from Congenital Hearing Loss

The final cost element in our analysis was the monetary value of DALYs lost due to hearing loss. Using the human capital approach we attached a monetary value to DALY estimates. DALYs consist of the number of years lived with hearing loss (YLD) and the number of years of life lost (YLL) due to premature mortality.^42^ YLL were estimated by multiplying the number of years lived with hearing loss for each person by the disability weight for different levels of hearing loss severity—0.01 for mild, 0.03 for moderate and 0.20 for severe.^43^ We estimated the monetary value of DALYs lost due to hearing loss as follows:

$$\mathrm{TMVD}=\left\{\left[\begin{array}{l} \mathrm{GDP}\;\mathrm{per}\;\;\mathrm{capita}\;\;2022-\\ \mathrm{CHE}\;\;\mathrm{per}\;\mathrm{capita}\;\;2022 \end{array}\right]\;\times \;\mathrm{DALY}\right\}44$$

Where TMVD is the total monetary value of DALYs due to hearing loss and CHE is the current health expenditure per capita.

### Sensitivity Analysis

Our research was based on a range of assumptions and sources, with some resulting in uncertainty in the estimated economic costs of hearing loss. For the univariate sensitivity analysis, we varied model parameters using estimates provided for in the literature^42^ and where unavailable by ±50%. To assess the robustness of our final cost estimates to uncertainty in multiple inputs, we conducted a probabilistic sensitivity analysis (PSA). We fit appropriate probability distributions to each input and ran 1000 Monte Carlo simulations that drew randomly from the distributions.^45^ The parameters considered in the PSA included initial screening coverage, prevalence of congenital hearing loss by degree of severity, the cost of hearing aids, cochlear implant coverage and special education and the unemployment rate.

## Results

We estimated the costs of hearing loss for a birth cohort of 998 362 children born in 2022. Approximately 3994 live births were estimated to have congenital hearing loss. We assumed the audiological profile of the cohort was the same as that of a previous study of children with hearing loss in South Africa and that it would remain consistent throughout their lifetime (see Table 2).^46^

**Table 2.** Audiological Profile of South African Population with Congenital Hearing Loss Estimates in 2022.

Degree of hearing loss	Number of cases
Mild hearing loss	180
Moderate hearing loss	694
Severe hearing loss	1243
Profound hearing loss	1877
Total	3993

### Direct Health Care Costs

Table 3 summarizes the direct healthcare costs, cost per person, as well as the percentage distributions of the cost categories. Direct health care costs to the South African government were estimated at ZAR 383 billion (US 24 million) for the cohort from birth to 64 years of age. Hearing aid fitting, maintenance and replacement made up the largest portion of total discounted healthcare costs at 78% of total costs. Specialist visits made up 5% of total costs and cochlear implant costs made up 8% of total healthcare costs.

**Table 3.** Discounted Direct Healthcare Costs.

Cost category	Total healthcare costs (ZAR)	Cost/person	% of total
Newborn screening	7 568 506.19	75.81	1.98
Facility based follow up testing	18 006 640.60	406.85	4.70
ABR testing	2 422 258.78	922.89	0.63
Hearing aids fitting, maintenance and replacements	300 104 475.40	404 756.64	78.34
Cochlear implants	32 022 346.13	1 706 901.33	8.36
Rehabilitative services	1 065 839.64	66 160.13	0.28
Specialist visits	19 480 690.32	13 897.90	5.08
Medication to treat Otitis Media	2 430 367.90	605.59	0.63
Total	383 101 124.96		100.00

**Figure 1.**
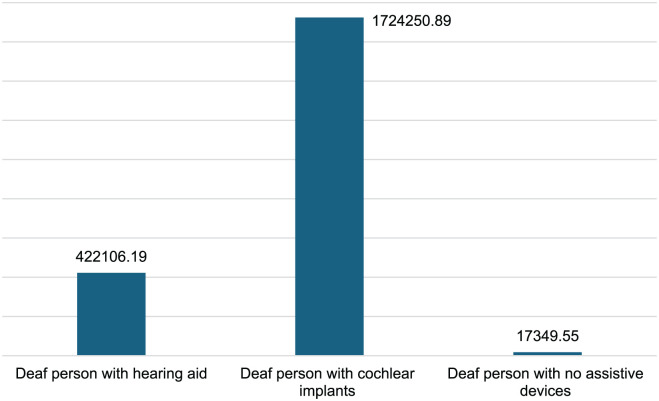
Total lifetime healthcare costs per person in ZAR.

Figure 1 shows the total lifetime healthcare costs associated with hearing loss. Individuals with cochlear implants had the highest lifetime costs at ZAR 1.7 million (approximately USD 106 thousand). Hearing aid users’ lifetime costs were approximately 24% of the costs of cochlear implant users. Individuals with no assistive devices have the least cost at an estimated ZAR 17.3 thousand (USD 1 thousand) per lifetime.

Table 4 shows the total lifetime and per person societal education, social assistance and lost productivity costs for the cohort.

**Table 4.** Total Lifetime and Per Person Societal Costs Per Cost Element.

Cost element	Total lifetime economic cost ZAR (USD)	Cost/person
Education	4 007 167 108 (248 737 871.40)	1 003 435.40 (62 286.49)
*Social assistance*		
Care dependency	69 719 229.20 (4 327 698.89)	314 084.71 (19 496.26)
Disability grant	38 954 080.61 (2 418 006.25)	
*Productivity losses*		
Unemployment	4 240 307 856.40 (263 209 674.51)	3 117 124.25 (193 490.04)
Reduced productivity	7 639 381 714.75 (474 201 223.76)	

### Education Costs

Special schooling cost was estimated at approximately ZAR 4 billion (USD 248.7 million) for 12 years of education. The average cost of special education per child with hearing loss was approximately ZAR 1.2 million (USD 77 thousand).

### Social Assistance Costs

Total discounted social assistance costs were estimated at ZAR 108.7 million (USD 6.7 million) for the cohort. Total care dependency costs were an estimated ZAR 69.7 million (USD 4.3 million), and disability grant costs were estimated at ZAR 39 million (USD 2.4 million).

### Monetary Value of Disability Adjusted Life Years Lost from Congenital Hearing Loss

**Figure 2.**
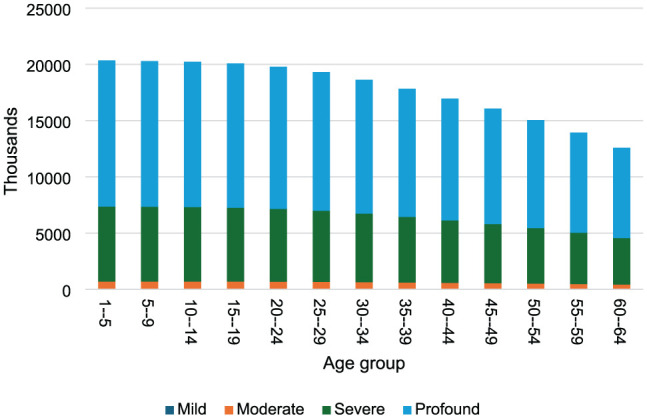
Monetary value of DALYs lost by age group and severity of hearing loss.

The total monetary value attached to DALYs lost due to congenital hearing loss was approximately ZAR 231.2 million (USD 14.3 million). Profound hearing loss has the highest loss making up 64% (ZAR 147.6 million to USD 9.2 million) of all DALY monetary losses. Mild hearing loss had the least at 0.3% (ZAR 693.6 thousand—USD 43.1 thousand) of DALY monetary losses (Figure 2).

### Productivity Losses

**Figure 3.**
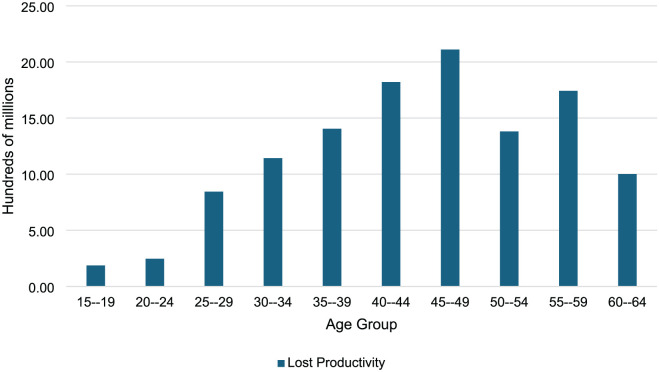
Adjusted productivity losses by age group.

Total discounted productivity losses due to moderate to severe hearing loss over the lifetime of the cohort due to unemployment and reduced productivity were approximately ZAR 4.2 billion and ZAR 7.6 billion, respectively totaling ZAR 11.9 billion (USD 737.4 million). Figure 3 shows the adjusted productivity losses discounted to 2022 values by age group. Productivity losses increased with age peaking between the ages of 45 to 49 years. Losses in this age range made up 18% of all productivity losses during the lifetime of the cohort. Productivity losses were at their lowest in the youngest age group 15 to 19 years old at ZAR 186.3 million (USD 11.6 million). The estimated productivity loss per individual with moderate to severe hearing loss over the course of their life was approximately ZAR 3 million (USD 184.7 million). Productivity loss figures by age can be found in Supplemental File 1, Tables 2 and 3.

### Total Economic Costs

Table 5 shows the total discounted economic cost to society attributable to hearing loss. Total economic cost was R 16.4 billion (USD 1 billion) accrued over 64 years or R 255.9 million (USD 15.9 million) per year for the cohort. Total discounted cost per person with hearing loss was R 4.1 million (USD 254.6 thousand) per lifetime.

**Table 5.** Total Lifetime Economic Costs for the Cohort.

Total economic cost	ZAR 16 378 631 114.25 (USD 1 016 674 805)
Total economic cost per year	ZAR 255 916 111.20 (USD 15 885 543.83)
Total cost per person	ZAR 4 101 375.83 (USD 254 585.71)

Productivity losses made up the largest proportion of societal costs with losses of 72% of total costs. Education costs were 24% of total societal economic costs and direct healthcare costs made up approximately 2.3% of total costs incurred. The monetary value of DALYs lost due to hearing loss made up 1.4% of total economic costs and social support had the least cost component of less than 1% of total economic costs.

### Sensitivity Analysis

**Figure 4.**
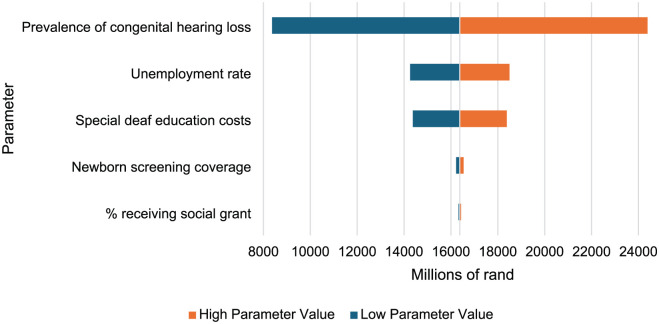
One way sensitivity analysis.

Results of one-way sensitivity analyses for total discounted societal economic costs are presented in Figure 4. A ±50% change in prevalence of congenital hearing loss would have the greatest potential impact on overall societal costs. Social assistance costs had the least impact on total societal cost.

**Figure 5.**
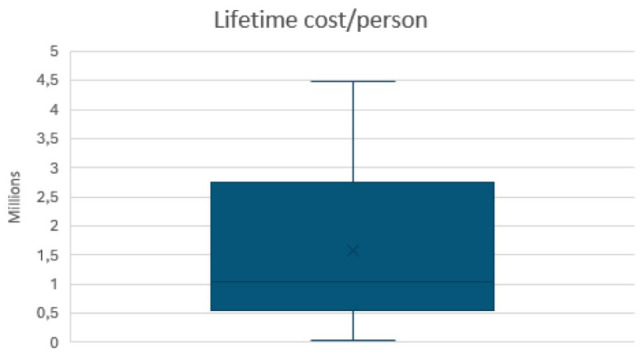
PSA showing the range of average cost per person over their lifetime.

The results of the distribution of cost per person per lifetime from the PSA is depicted in the box plot (Figure 5). The median cost was approximately ZAR 1.1 million (USD 64.5 thousand), with an interquartile range spanning from approximately ZAR 560.6 thousand to ZAR 2.7 million (USD 163.7 thousand), demonstrating substantial variability in individual costs. The mean cost, (“X”), was higher than the median, suggesting a positively skewed distribution driven by higher-cost outliers. The overall range of costs observed in the sample ranged from approximately ZAR 265 thousand (USD 1.5 thousand) per person per lifetime to approximately ZAR 4.5 million (USD 279 thousand).

## Discussion

We estimated the total discounted economic costs of hearing loss for a cohort of individuals in South Africa with 2022 as our base year. We considered costs in the healthcare and education sector, productivity losses due to unemployment and underemployment as well as the monetary value of DALY losses. Our model considered the societal perspective and a 64-year time horizon. Our analysis found that lifetime total societal economic costs were approximately ZAR 16.4 billion (USD 1 billion). Cost estimates per person were approximately ZAR 4 million (USD 256 thousand). This estimate was mostly made up of productivity losses (73% of total costs). The country would lose an estimated ZAR 11.9 billion (approximately US 737 million) in productivity due to congenital hearing loss during the lifetime of this cohort. Productivity losses could be attributed to the 57% wage differential between the non-disabled and disabled working population in South Africa and the unemployment rate.

There is a paucity of studies that estimate the societal costs of congenital hearing loss and non that have estimated costs of a cohort from birth to death in Africa. Schroeder et al^62^ estimated economic costs of congenital bilateral permanent childhood hearing impairment in a sample of children from Southern England between 2002 and 2003. They however only estimated the societal economic costs of hearing impairment in the preceding year of life for children aged 7 to 9 years of age. The study reported substantial increases in costs for their study sample compared to children with typical hearing. Similar to our study, they found that severity of hearing loss was an important predictor of societal costs in the preceding year of life.^62^

Mohr et al^63^ conducted a cohort-survival analysis to estimate the societal lifetime costs of hearing loss in the United States of America. In contrast to our study, their analysis included all incident cases occurring over the cohort’s lifetime and was restricted to individuals with severe and profound hearing loss. Consistent with our findings, they reported that reduced productivity represented a major component of total economic costs, contributing approximately 60%.^63^ The inclusion of only severe cases likely resulted in higher average cost estimates, whereas our broader case definition provides a more comprehensive reflection of the total societal burden across the full spectrum of hearing loss severity.

Our study estimates are consistent with the literature on healthcare spending in low- and middle-income countries. High income countries allocate more than 7% of their GDP on healthcare and upper middle-income countries allocate an average of 2.9%. South Africa’s public healthcare spending is 3.27% of the country’s GDP. This difference highlights the need for LMICs to invest more in healthcare spending including prevention and management of hearing loss. Poor literacy outcomes and subsequent unemployment among individuals who are deaf or hard of hearing are often the result of insufficient early language support, rather than the hearing loss itself.^64^ These adverse outcomes are largely preventable through timely and appropriate early intervention.^65,66^ Moreover, social exclusion—fueled by misconceptions that equate deafness with disability—may further exacerbate barriers to education and employment.^67,68^ This suggests that the negative outcomes commonly associated with childhood hearing loss are primarily driven by structural and systemic factors rather than the impairment per se. Consequently, policy responses that prioritize early intervention and address social and structural inequities hold significant potential to mitigate long-term economic and social costs.

Our analysis highlights the need to address unemployment rates among the deaf population in South Africa. Reducing baseline unemployment rates would result in substantial cost savings. A 50% reduction in baseline unemployment rates resulted in a 12% reduction in total economic costs. Cost per person estimates dropped from the baseline estimate of approximately R 4.1 million (USD 850 thousand) per person to R 3.6 million (USD 222 thousand). Social assistance figures are not an exact estimate of the cost burden incurred because of hearing loss as social assistance grant disbursements are not disaggregated by type of disability. Information on disability specific social grant disbursements needs to be available to estimate accurate potential cost savings.

Our study found that health losses from DALYs are mediated by degree of hearing loss. The profoundly deaf are those with the greatest DALY losses and this is consistent across the whole life span of all age groups. Given that this work focused on the born deaf cohort and results are not confused by the effects of hearing loss with age like most international data, it points to the significance of working to prevent or reduce this predictive effect.

### Strengths and Limitations

The main strength of this study is that this is the first study in South Africa that has attempted to quantify the lifetime economic costs of congenital hearing loss. The results of this study could help inform the development of policies and inform resource allocation decisions to support services, healthcare, education and employment programs. The second strength of our study is that we have estimated the costs of hearing loss over a lifetime. Unlike prevalence studies, this approach is a strength as it captures all the cost required for people with hearing loss from birth until death. The third strength is that unlike other international studies of economic impact of hearing loss, our study has exclusively focused on the implications of early deafness, rather than considering the economic arguments for adult and age-related hearing loss which are very different populations.

Our main limitation is that our study most likely underestimates the true societal costs of hearing loss in South Africa due to assumptions about the model parameters. Firstly, analysis was done using prevalence data which is not South Africa specific. No comprehensive, nationwide studies on the prevalence of hearing loss in South Africa have been done and it was assumed that the incidence of congenital hearing loss to be the same as the global prevalence rate of moderate to higher grades of hearing loss for the post neonatal stage (28-364 days).^10^ We also assumed that hearing loss would not become worse over the lifetime of the cohort, omitting costs associated with degenerative hearing loss over time. We, however, used local estimates for the hearing loss profile.^46^

Secondly, the offer and take up of newborn screening is very low in hospitals (10%); it is not a universal health provision. Only 35% of individuals referred for further audiological testing present themselves for further investigation. Most newborn deaf children in any given year will not be identified, therefore the figures used in the analysis underestimate the number of congenitally deaf children born in 2022. Direct healthcare costs associated with hearing loss are likely to be higher than our estimates.

Third, education costs did not include an estimate of early intervention costs which are not health/audiological related such as the costs of family support and language development programs in the first few years of life which are recommended as a key component by Health Professions Council of South Africa and international guidelines on early childhood hearing loss.

People with disabilities had a two-fold higher mortality rate than people without disabilities in LMICs but we could not find any data that was specific to the South African deaf population.^69^ We therefore assumed that survival probability for individuals with hearing loss was the same as that of individuals without hearing loss.

Due to data limitations, the real value of productivity lost is likely to be an underestimation. Our study used unemployment estimates that are likely lower than real levels experienced. Unemployment estimates for deaf people in South Africa are reported to be as high as 80% compared to 39% in the general population used in our analysis, but there are no studies to support these assertions. In addition, we did not factor in economic losses incurred by caregivers, productivity losses due to absenteeism and presenteeism, tax rebates afforded for disability related expenses and any out-of-pocket expenses incurred because of hearing disability.

We could not find any data relating to the proportion of people with hearing loss who receive social assistance, and our estimate was based on social assistance for all disabled persons in South Africa.^52^

## Conclusion

Hearing loss not only affects the physical aspects of a person’s life but has far reaching societal and economic implications. Our study results demonstrate the “hidden” costs associated with hearing loss that are not apparent to policy makers as the focus is mostly on healthcare related costs. Understanding the costs of hearing loss provides important information for identifying areas of need for future research and healthcare investment. The results show the need to invest in early identification and intervention mechanisms for hearing loss to avert incurring costs further down the line in the form of lost productivity due to unemployment or underemployment. These cost estimates also provide an important benchmark and baseline data for cost-effectiveness analyses for interventions for addressing hearing loss in South Africa.

## Supplemental Material

sj-docx-1-his-10.1177_11786329251401414 – Supplemental material for The Economic Costs of Congenital Hearing Loss in a South African Cohort Supplemental material, sj-docx-1-his-10.1177_11786329251401414 for The Economic Costs of Congenital Hearing Loss in a South African Cohort by Winfrida Tombe-Mdewa, Claudine Storbeck, Alys Young, Aisha Moolla, Susan Goldstein and Evelyn Thsehla in Health Services Insights
