# Supplementary material for: The Economic Costs of Congenital Hearing Loss in a South African Cohort
Source: Health Serv Insights. 2026 Mar 19;19:11786329251401414. doi: 10.1177/11786329251401414 (PMC13009870; doi:10.1177/11786329251401414)
Supplement: sj-docx-1-his-10.1177_11786329251401414 – Supplemental material for The Economic Costs of Congenital Hearing Loss in a South African Cohort [file sj-docx-1-his-10.1177_11786329251401414.docx]

Appendix 1

Table 1: Model parameter assumptions

|  | Assumptions |
| --- | --- |
| Newborn screening | Screening in the form of otoacoustic emissions (OAE) screening  Done at discharge by audiologist  Infants who fail to pass the test are referred for appropriate audiologic and medical evaluations to confirm the presence of hearing loss  10% of newborns are screened at birth |
| Follow up screening for infants that fail the newborn screening  Diagnostic Assessments | Otoscopic examination, a tympanogram and a repeat OAE conducted by an audiologist  If the results of the rescreening procedures are inconclusive, the infant is referred for diagnostic ABR measures  35% of patients referred for further screening from hospitals or schools follow through ^72^  10% referred for auditory brain response testing (ABR) |
| Auditory brainstem response testing | Theatre procedure done by paediatric audiologist and anesthetist |
| Hearing aids fitting, maintenance and replacements | Done by audiologist at the hospital  Five consultations needed, initial consult for audiological assessment, fitting of the hearing aid and three follow up consultations  All infants that have bilateral hearing losses are fitted with 2 hearing aids  Hearing aids are replaced every 4 years  Hearing aid and earmolds are replaced as follows:  Year 1 – 12 times  Year 2 – 6 times  Year 3 to Year 5 – 4 times  Year 5 to year 18 -2 times  Above 18 – once every 3 years  52 batteries per patient are needed each year ^73^ |
| Cochlear implants | Costs included in the estimation:  Cost of device  Costs of surgery   \| Post-operative costs and cochlear implant mapping \| \| --- \|  \| Rehabilitation costs \| \| --- \|   Maintenance (Repairs and upgrade of the processor every 7 years) |
| Rehabilitative services | Inclusive of speech therapy and aural habilitative sessions  6 sessions of each on issue of a new hearing aid  Done by a speech therapist |
| Specialist Visits | 4 specialist visits per year from 0 to 6 years  2 specialist visit per year from 6 to 14 years  1 specialist visit from 15 to 64 years |

Table 2: Productivity losses by age group - Unemployment

| Loss Due to Unemployment | Age group | Lost Productivity | % of total |
| --- | --- | --- | --- |
|  | 15--19 | 137218096,40 | 3% |
|  | 20--24 | 181184714,00 | 4% |
|  | 25--29 | 441500237,90 | 10% |
|  | 30--34 | 580241881,30 | 14% |
|  | 35--39 | 577558373,40 | 14% |
|  | 40--44 | 748727250,40 | 18% |
|  | 45--49 | 671455977,80 | 16% |
|  | 50--54 | 284932101,20 | 7% |
|  | 55--59 | 359451832,20 | 8% |
|  | 60--64 | 258037392,00 | 6% |
|  |  | 4240307856,40 | 100% |

Table 2 : Productivity loss by age group - Reduced productivity

| Loss due to reduced productivity | Age group | Lost Productivity | % of Total Losses |
| --- | --- | --- | --- |
|  | 15--19 | 49083596,08 | 1% |
|  | 20--24 | 64810674,05 | 1% |
|  | 25--29 | 403179943,84 | 5% |
|  | 30--34 | 562238337,57 | 7% |
|  | 35--39 | 827029628,20 | 11% |
|  | 40--44 | 1072133394,80 | 14% |
|  | 45--49 | 1438859389,20 | 19% |
|  | 50--54 | 1096063246,18 | 14% |
|  | 55--59 | 1382722201,14 | 18% |
|  | 60--64 | 743261303,70 | 10% |
|  |  | 49083596,08 | 1% |
